# Secondary sclerosing cholangitis in critically ill patients with febrile infection-related epilepsy syndrome (FIRES): a case series

**DOI:** 10.3389/fneur.2025.1557377

**Published:** 2025-04-24

**Authors:** Lorenzo Muccioli, Lidia Di Vito, Elena Pasini, Lorenzo Ferri, Giovanni Vitale, Alessandro Granito, Barbara Mostacci, Manuel Moneti, Laura Licchetta, Rocco Liguori, Paolo Tinuper, Carlo Alberto Castioni, Francesca Bisulli

**Affiliations:** ^1^Department of Biomedical and Neuromotor Sciences, University of Bologna, Bologna, Italy; ^2^IRCCS Istituto delle Scienze Neurologiche di Bologna, Full Member of the ERN EpiCARE, Bologna, Italy; ^3^Internal Medicine Unit for the Treatment of Severe Organ Failure, IRCCS Azienda Ospedaliero-Universitaria di Bologna, Bologna, Italy; ^4^Division of Internal Medicine, Hepatobiliary and Immunoallergic Diseases, IRCCS Azienda Ospedaliero-Universitaria di Bologna, Bologna, Italy

**Keywords:** FIRES, status epilepticus, NORSE, intensive care, ketamine, drug-induced liver injury (DILI)

## Abstract

**Objectives:**

To describe the occurrence of secondary sclerosing cholangitis in critically ill patients (SC-CIP) with febrile infection-related epilepsy syndrome (FIRES).

**Methods:**

Monocentric retrospective analysis of all adult patients with FIRES admitted from January 2020 to December 2024.

**Results:**

Four patients (3 males) with a mean age of 24 years (range: 18–40 years) and no significant medical history presented with cryptogenic FIRES. They required treatment with antiseizure medications (mean: 9; range: 8–10), anesthetics (propofol, midazolam and ketamine in all cases), and immunotherapies. The average duration of status epilepticus (SE) was 57 days (range: 34–90 days), while the mean duration of intensive care unit (ICU) stay was 82 days (range: 58–117 days). All patients developed cholestatic liver disease during their ICU stay, reversible in one case. In the three cases with persistent injury (75%), SC-CIP was diagnosed with MR-colangiography after a mean of 106 days from SE onset.

**Discussion:**

The high incidence of SC-CIP in our cohort of patients with FIRES suggests a link between these two rare conditions, likely related to prolonged intensive care, hyperinflammation and polytherapy, including ketamine use. Vigilant monitoring of liver disease progression in critically ill patients with FIRES and similar predisposing factors may allow early recognition of SC-CIP and improved patient outcomes.

## Introduction

Febrile infection-related epilepsy syndrome (FIRES) is a rare form of cryptogenic new-onset refractory status epilepticus (NORSE) characterized by a febrile illness starting before the onset of SE (1). The exact pathogenesis remains unclear; however, it is believed to involve a dysregulated systemic inflammatory response following infection, which can result in blood–brain-barrier dysfunction and trigger an uncontrolled cytokine-mediated neuroinflammatory cascade, promoting the development of seizures and their refractoriness (1, 2).

Sclerosing cholangitis (SC) is a rare chronic cholestatic biliary disease characterized by inflammation and fibrosis of the bile ducts, leading to strictures and destruction of the biliary tree, which can result in biliary cirrhosis (3). SC may be classified into primary and secondary forms. Primary SC is a presumed progressive autoimmune condition linked to inflammatory bowel disease and antineutrophilic cytoplasmic antibodies (ANCA), which can be complicated by cholangiocarcinoma (3). Secondary SC arises from identifiable injuries to the biliary system, such as prolonged biliary obstruction, infections, surgical trauma, or ischemia (3, 4). A newly recognized entity, SC in critically ill patients (SC-CIP), is characterized by rapid progression and has been associated with ketamine use (3, 5, 6).

To date, there has been no established link between FIRES and SC. In this study, we present a case series of patients with FIRES and describe their association with SC-CIP.

## Methods

We conducted a retrospective analysis of all adult patients with FIRES admitted to our Institute from January 2020 to December 2024. FIRES diagnosis was established based on the international consensus definition, i.e., a clinical presentation of NORSE with a prior febrile infection starting between 2 weeks and 24 h prior to onset of refractory SE, with or without fever at onset of SE (1). Data were systemically extracted from electronic health records and included demographic information, clinical features, laboratory and imaging studies, treatment interventions, and patient outcomes. The diagnosis of SC was based on magnetic resonance (MR)-colangiopancreatography findings, on clinical history and, according to the European Association for the Study of the Liver (EASL) guidelines by the exclusion of chronic obstructive, immune-mediate, infectious, and hereditary etiologies (3). Patients’ data were extracted from electronic health records. Informed written consent was obtained from all patients for participation in the study.

## Results

Four patients (three males) with a mean age of 24 years (range: 18–40 years) and no significant past medical history presented with FIRES. The key features are summarized in Table 1. Fever preceded the onset of generalized convulsive refractory SE by an average of 5 days (range: 3–6 days) in all patients.

**Table 1:** Demographic, clinical and laboratory features of the patients.

	Case 1	Case 2	Case 3	Case 4
Age at onset, sex	20 yo, male	40 yo, female	18 yo, male	19 yo, male
Ethnicity	Caucasian	Caucasian	Asian	Caucasian
Follow-up	33 months	48 months	14 months	30 months
Status epilepticus				
Antiseizure medications	10 (LEV, LAC, PHT, PER, BRV, PB, ZNS, VPA, CBD, CLB)	10 (LEV, LAC, PHT, PER, BRV, PB, ZNS, VPA, TPM, CLB)	9 (LEV, LAC, PHT, PER, BRV, PB, VPA, CBD, CLB)	8 (LEV, LAC, PHT, PER, BRV, PB, VPA, TPM)
Anesthetics	PRO, MDZ, KET	PRO, MDZ, KET, TPS	PRO, MDZ, KET	PRO, MDZ, KET
Immunotherapies (day of first administration)	MP (+3), PLEX (+4), IVIg (+8), anakinra (+33)	MP (+23), IVIg (+9), RTX (+31)	MP (+1), IVIg (+4), CP (+11)	MP (+5), PLEX (+12), IVIg (+19), CP (+21)
Other therapies for SE	VNS, KD	None	VNS	None
SE duration	90 days	45 days	34 days	59 days
ICU stay	117 days	58 days	84 days	69 days
Relapse	Yes, at 12 months	No	No	Yes, at 12 months
mRS at last follow-up	2	2	4	2
Ketamine doses				
Maximal dose	3.5 mg/kg/h	5.3 mg/kg/h	2.0 mg/kg/h	5.0 mg/kg/h
Treatment duration	22 days	15 days	15 days	40 days
Total ketamine load	135.8 g	103.8 g	44.2 g	330.0 g
Ketamine load pro kg	1,636 mg/kg	1,383 mg/kg	539 mg/kg	3,402 mg/kg
Hepatobiliary assessments				
Sclerosing cholangitis	No	Yes	Yes	Yes
MR-colangiography timing after SE onset	Not performed	49 days	75 days	194 days
Peak bilirubin levels (direct, indirect)*	Normal	4.7 mg/dL (2.3, 2.5)	7.2 mg/dL (4.4, 2.8)	13.6 mg/dL (7.3, 6.4)
Peak GGT levels*	910 U/L	1,485 U/L	4,201 U/L	3,350 U/L
Peak ALP levels*	167 U/L	748 U/L	1760 U/L	1,000 U/L
Peak AST levels*	Normal	135 U/L	324 U/L	299 U/L
Peak ALT levels*	Normal	305 U/L	738 U/L	200 U/L
Liver abscesses	No	Yes	No	Yes

^*^Reference laboratory values: bilirubin total <1.2 mg/dL (direct <0.3, indirect <0.9); GGT <38 U/L; ALP <120 U/L; AST < 35 U/L; ALT <35 U/L.

ALP, alkaline phosphatase; ALT, alanine aminotransferase; AST, aspartate aminotransferase; BRV, brivaracetam; CBD, cannabidiol; CLB, clobazam; CNZ, clonazepam; CP, cyclophosphamide; IVIg, intravenous immunoglobulin; KD, ketogenic diet; KET, ketamine; LAC, lacosamide; LEV, levetiracetam; MP, methylprednisolone; mRS, modified Rankin Scale; PB, phenobarbital; PER, perampanel; PHT, phenytoin; PRO, propofol; RTX, rituximab; SE, status epilepticus; TPM, topiramate; TPS, sodium tiopenthal; VNS, vagus nerve stimulation; ZNS, zonisamide.

Etiology was cryptogenic despite an extensive diagnostic work-up, including anti-neuronal autoantibody testing, infectious disease testing, metabolic and toxicology screenings, tumor screening by whole body PET-CT scans and thoraco-abdominal CT scans.

Brain MRI in the acute stage showed a combination of T2-hyperintentisities and swelling in the following regions: claustrum (*n* = 3), mesial temporal lobe (*n* = 2), cingulate cortex (*n* = 2), pulvinar (*n* = 2), frontal lobe (*n* = 1).

Patients required treatment with several antiseizure medications (ASM) (mean: 9; range: 8–10), additional antiseizure therapies (ketogenic diet, *n* = 1; vagus nerve stimulation, *n* = 2), anesthetics (propofol, midazolam, and ketamine, *n* = 4 [doses detailed in Table 1]; pentothal, *n* = 1), and immunotherapies (methylprednisolone and IVIg, *n* = 4; plasmapheresis, *n* = 2; anakinra, *n* = 1; rituximab, *n* = 1; cyclophosphamide, *n* = 2). The average duration of SE was 57 days (range: 34–90 days), while the mean duration of ICU stay was 82 days (range: 58–117 days). Three patients experienced persistent hyperpyrexia refractory to high-dose antipyretic medications (paracetamol, diclofenac) throughout the ICU stay, despite targeted antibiotic therapies to concomitant ICU-related bacterial septicemia and pneumonia. Case 2 had long-lasting hypogammaglobulinemia following treatment with rituximab (1,000 mg, two doses in 14 days), requiring monthly immunoglobulin replacement therapy. Case 3 developed severe paraparesis likely related to a spinal cord ischemic injury occurred during the ICU stay.

In two cases (1 and 4), convulsive RSE relapsed after 12 months. In case 1, the relapse was not preceded by fever and resolved after 30 days following treatment with cyclophosphamide; the brain MRI showed new lesions of bilateral claustrum and pulvinar in the acute phase. In case 4, it was triggered by an acute tonsillitis and resolved after 5 days without using immunotherapies; brain MRI did not show any new lesions.

At the last follow-up, which averaged 31 months, all patients had drug-resistant epilepsy and cognitive deficits of varying severities. The mean modified Rankin scale was 2.5 (range: 2–4).

**Figure 1:**
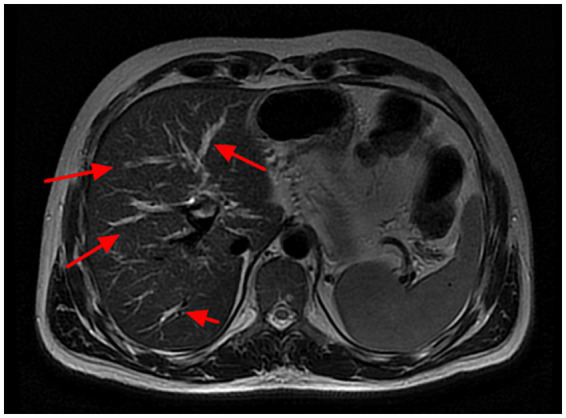
Magnetic resonance colangiography findings in case 2. The red arrows indicate dilated bile ducts alternating with stenosis, findings consistent with sclerosing cholangitis.

All patients developed cholestatic liver disease during their ICU stay, reversible in case 1 after reduction of ASMs, notably valproate and phenobarbital. In the three cases with persistent injury, SC involving intra- and extrahepatic bile ducts was diagnosed with MR-colangiography after a mean of 106 days from SE onset and was classified as SC-CIP (Figure 1). ANCA and anti-IgG4 antibodies were negative. Liver blood tests were within range at admission. During the hospitalization, these were altered up to (mean ± SD): total bilirubin 8.5 ± 3.7 mg/dL (direct>indirect), gamma-glutamyltransferase (GGT) 3,012 ± 1,134 U/L, alkaline phosphatase (ALP) 1,169 ± 430 U/L, AST 220 ± 78 U/L, ALT 414 ± 232 U/L (Table 1); coagulation tests were normal. Cholangitis was complicated by hypercholesterolemia (*n* = 2) and liver abscesses (*n* = 2). All three cases are being treated with ursodeoxycholic acid (15–20 mg/kg/d) and bezafibrate, helpful in alleviating hepatobiliary injury (7), and have preserved liver function at their last follow-up.

Case 1 was found to have intrahepatic bile duct dilation on ultrasonography without SC. Despite normal laboratory exams, he remains under gastroenterological follow-up and is also being treated with ursodeoxycholic acid.

## Discussion

In our cohort of adult patients with FIRES, three out of four (75%) developed SC, raising questions about a potential association between these two rare conditions that has not been previously documented.

Patients with FIRES, including those in our study, require prolonged intensive care treatment, a setting where secondary SC-CIP has been recently observed with increased frequency, nevertheless remaining ultrarare (approximately 250–300 cases reported) (5). The pathogenesis of SC-CIP is characterized by ischemic colangiopathy in combination with changes in bile composition, that lead to necrosis of cholangiocytes and bile cast formation, resulting in progressive destruction and obliteration of bile ducts (4, 5). While critical illness and mechanical ventilation may contribute to biliary ischemia, the hyperinflammatory state typical of FIRES might result in downregulation of hepatobiliary transporters and formation of toxic bile (5, 8).

Additionally, polytherapy with ASMs, anesthetics and antipyretics at high doses over extended periods can lead to the development of drug-induced liver injury (DILI). Although DILI might be transient and resolve after drug withdrawal or dose adjustments, as observed in case 1, in a minority of patients, it can lead to bile duct injury that is visualized on imaging as SC-like changes (9). Ketamine is increasingly being used for the treatment of RSE (10) and has been implicated in cholangiopathy, particularly in cases of chronic abuse (11). Accordingly, the EASL Clinical Practice Guidelines on SC includes ketamine use in the differential diagnosis of SC forms (3). Its intravenous use in the ICU has been associated with SC also in critically ill COVID-19 patients (12), a condition sharing a hyperinflammatory profile with FIRES (2). While recent forensic toxicology data challenge the notion of ketamine as a primary causative agent in SC-CIP (6), it may act as a contributing factor in patients with a previously injured biliary tract due to concomitant factors, particularly when given at high doses as in our cohort.

The laboratory features of SC-CIP observed in our patients are consistent with established findings, including rapidly rising levels of GGT and ALP, along with elevated bilirubin levels - although less pronounced - while serum levels of aminotransferases are usually only mildly increased (4).

As the clinical signs during the initial phase of SC-CIP are nonspecific, diagnosis is often overlooked and SC-CIP prevalence underestimated (4, 5). This highlights the necessity for vigilant monitoring of liver disease progression in patients with FIRES and underscores the importance of requesting a careful work-up for the differential diagnosis of SC and a MR-cholangiography in cases exhibiting persistent cholestatic injury. This vigilant approach should also be maintained in critically ill patients without FIRES who share the predisposing factors present in our cohort. Timely diagnosis is crucial, as SC-CIP carries a dismal prognosis characterized by high mortality and rapid progression to liver cirrhosis (4). Medical treatment with ursodeoxycholic acid is commonly used but has limited efficacy, and endoscopic interventions allow only for palliative treatment; hence, early referral for liver transplantation should be considered for selected cases (4, 5).

We acknowledge some limitations of our study. First, our cohort consists exclusively of adult patients, even though FIRES is more commonly described in pediatric populations. While all our cases met the diagnostic criteria for FIRES, further research is needed to determine whether SC also occurs in younger FIRES patients. Second, while our findings suggest a potential link between SC and NORSE/FIRES, we cannot exclude that SC-CIP may primarily be a complication of prolonged ICU treatment rather than a distinct feature of NORSE/FIRES itself. However, it is noteworthy that during the same study period (2020–2024), none of the other critically ill patients in our neuro-ICU developed SC-CIP, suggesting that unique factors in this population may contribute to its development. As mentioned above, these factors include the intensive polytherapy regimens typically required in FIRES, including prolonged ketamine exposure and the hyperinflammation characteristic of the condition.

## Conclusion

Our findings suggest that patients with FIRES may be at an increased risk of developing SC-CIP, likely due to prolonged intensive care, hyperinflammation, and polytherapy with ASMs and anesthetics. Neurologists, intensivists and gastroenterologists should be aware of this possible association, as timely diagnosis of SC-CIP could significantly impact patient prognosis. Further research is needed to corroborate our findings and to clarify the role of ketamine in the pathogenesis of SC-CIP, as this may have clinical implications in the management of RSE.

## Data Availability

The original contributions presented in the study are included in the article/supplementary material, further inquiries can be directed to the corresponding author.

## References

[1] Hirsch LJ Gaspard N van Baalen A Nabbout R Demeret S Loddenkemper T. Proposed consensus definitions for new-onset refractory status epilepticus (NORSE), febrile infection-related epilepsy syndrome (FIRES), and related conditions. Epilepsia. (2018) 59:739–44. doi: 10.1111/epi.14016, PMID: 29399791

[2] Pensato U Muccioli L Cani I Janigro D Zinzani PL Guarino M. Brain dysfunction in COVID-19 and CAR-T therapy: cytokine storm-associated encephalopathy. Ann Clin Transl Neurol. (2021) 8:968–79. doi: 10.1002/acn3.51348, PMID: 33780166 PMC8045903

[3] European Association for the Study of the Liver. Electronic address: easloffice@easloffice.eu; European Association for the Study of the liver. EASL clinical practice guidelines on sclerosing cholangitis. J Hepatol. (2022) 77:761–806. doi: 10.1016/j.jhep.2022.05.011, PMID: 35738507

[4] Ruemmele P Hofstaedter F Gelbmann CM. Secondary sclerosing cholangitis. Nat Rev Gastroenterol Hepatol. (2009) 6:287–95. doi: 10.1038/nrgastro.2009.46, PMID: 19404269

[5] Martins P Verdelho MM. Secondary Sclerosing cholangitis in critically ill patients: an underdiagnosed entity. GE Port J Gastroenterol. (2020) 27:103–14. doi: 10.1159/000501405, PMID: 32266307 PMC7113589

[6] Leonhardt S Jürgensen C Frohme J Grajecki D Adler A Sigal M. Hepatobiliary long-term consequences of COVID-19: dramatically increased rate of secondary sclerosing cholangitis in critically ill COVID-19 patients. Hepatol Int. (2023) 17:1610–25. doi: 10.1007/s12072-023-10521-0, PMID: 37119516 PMC10148013

[7] de Vries E Bolier R Goet J Parés A Verbeek J de Vree M. Fibrates for itch (FITCH) in Fibrosing Cholangiopathies: a double-blind, randomized, placebo-controlled trial. Gastroenterology. (2021) 160:734–743.e6. doi: 10.1053/j.gastro.2020.10.001, PMID: 33031833

[8] Yang H Plösch T Lisman T Gouw ASH Porte RJ Verkade HJ. Inflammation mediated down-regulation of hepatobiliary transporters contributes to intrahepatic cholestasis and liver damage in murine biliary atresia. Pediatr Res. (2009) 66:380–5. doi: 10.1203/PDR.0b013e3181b454a4, PMID: 19581828

[9] Ahmad J Rossi S Rodgers SK Ghabril M Fontana RJ Stolz A. Sclerosing cholangitis-like changes on magnetic resonance cholangiography in patients with drug induced liver injury. Clin Gastroenterol Hepatol. (2019) 17:789–90. doi: 10.1016/j.cgh.2018.06.035, PMID: 29966706 PMC6311440

[10] Yan M Sun T Liu J Chang Q. The efficacy and safety of ketamine in the treatment of super-refractory status epilepticus: a systematic review. J Neurol. (2024) 271:3942–52. doi: 10.1007/s00415-024-12453-7, PMID: 38782798 PMC11233303

[11] Schep LJ Slaughter RJ Watts M Mackenzie E Gee P. The clinical toxicology of ketamine. Clin Toxicol (Phila). (2023) 61:415–28. doi: 10.1080/15563650.2023.2212125, PMID: 37267048

[12] de Tymowski C Dépret F Dudoignon E Legrand M Mallet V Keta-Cov Research Group. Ketamine-induced cholangiopathy in ARDS patients. Intensive Care Med. (2021) 47:1173–4. doi: 10.1007/s00134-021-06482-3, PMID: 34313797 PMC8315088

